# Which risk factors are associated with stomal recurrence after total laryngectomy for laryngeal cancer? A meta-analysis of the last 30 years^☆^

**DOI:** 10.1016/j.bjorl.2020.03.002

**Published:** 2020-04-11

**Authors:** Jiwang Liang, Xiangyu Zhu, Wei Zeng, Tao Yu, Fengqin Fang, Yuejiao Zhao

**Affiliations:** a Cancer Hospital of China Medical University, Liaoning Cancer Hospital & Institute, Department of Head and Neck Surgery, Shenyang, People's Republic of China; b Cancer Hospital of China Medical University, Liaoning Cancer Hospital & Institute, Department of General Medicine, Shenyang, People's Republic of China; c Cancer Hospital of China Medical University, Liaoning Cancer Hospital & Institute, Department of Medical Imaging, Shenyang, People's Republic of China

**Keywords:** Laryngeal cancer, Stomal recurrence, Total laryngectomy, Risk factor, Meta-analysis, Câncer de laringe, Recorrência estomal, Laringectomia total, Fator de risco, Meta-análise

## Abstract

**Introduction:**

Stomal recurrence is a troublesome complication after total laryngectomy. Despite a large number of studies having been performed, there is still controversy about which risk factors are most significant for the development of stomal recurrence.

**Objective:**

The objective of the present meta-analysis was to analyze the potential factors leading to stomal recurrence after total laryngectomy.

**Methods:**

PubMed, Web of Science, Cochrane Library, and Ovid databases were systematically searched using multiple search terms. Eighteen studies with 6462 patients were identified. The quality of evidence was assessed by The National Institute for Health and Clinical Excellence.

**Results:**

The results showed that, tumor subsite (supraglottic vs. subglottic, RR = 0.292, 95% CI 0.142–0.600, *p* = 0.001; glottic vs. subglottic, RR = 0.344, 95% CI 0.175–0.676, *p* = 0.002), T stage (RR = 0.461, 95% CI 0.286–0.742, *p* = 0.001), preoperative tracheotomy (RR = 1.959, 95% CI 1.500–2.558, *p* < 0.001) were the high-risk factors associated with the development of stomal recurrence.

**Conclusion:**

From the results of our study, tumor subsite, T stage and preoperative tracheotomy were the significant risk factors for stomal recurrence. Methodologically high-quality comparative investigations are needed for further evaluation.

## Resumo

**Introdução:**

A recorrência estomal pós-laringectomia total e é uma complicação de difícil manejo. Apesar de um grande número de estudos ter sido feito, ainda há controvérsias sobre quais fatores de risco são mais significativos para o seu desenvolvimento.

**Objetivo:**

O objetivo da presente meta-análise foi analisar os fatores potenciais para recorrência estomal após a laringectomia total.

**Método:**

As bases de dados PubMed, Web of Science, Cochrane Library e Ovid foram sistematicamente pesquisadas com vários termos de busca. Dezoito estudos com 6.462 pacientes foram identificados. A qualidade da evidência foi avaliada pelo *National Institute for Health and Clinical Excellence*.

**Resultados:**

Os resultados mostraram que o subsítio tumoral (supraglótico vs. subglótico, RR = 0,292, IC 95% 0,142 ± 0,600, *p* = 0,001; glótico vs. subglótico, RR = 0,344, IC 95% 0,175 ± 0,676, *p* = 0,002), estágio T (RR = 0,461, IC 95% 0,286 ± 0,742, *p* = 0,001), traqueotomia pré-operatória (RR = 1,959, IC 95% 1.500 ± 2.558, *p* < 0,001) foram os fatores de alto risco associados ao desenvolvimento de recorrência estomal.

**Conclusão:**

Com base nos resultados de nosso estudo, o subsítio do tumor, o estágio T e a traqueotomia pré-operatória foram fatores de risco significativos para recorrência estomal. São necessárias investigações comparativas metodologicamente de alta qualidade para maior avaliação.

## Introduction

Laryngeal carcinoma represents one of the most common head and neck malignancies, accounting for approximately 20% of all cancer patients. Each year, approximately 10,000 persons in the US are diagnosed, accounting for 2.4% of new malignancies worldwide every year.^1^ The majority of cancers are squamous cell carcinoma, and up to 40% of cases present with advanced disease.^2^ Given the important role the larynx plays in human speech and communication, determining the optimal treatment of laryngeal cancer is critical. Laryngectomy has been a mainstay for treatment of laryngeal cancer for over 50 years. Although total laryngectomy (TL) offers significant therapeutic benefit, it is fraught with some long-term complications. The main postoperative complications after laryngectomy include laryngeal stenosis and pharyngo-peristomal fistula.^3^, ^4^ Stomal recurrence (SR) of laryngeal cancer is regarded as one of the most serious complications after TL. It is defined as “a diffuse infiltrate of neoplastic tissue at the junction of an amputated trachea and the skin”.^5^ According to the previous studies, the incidence rate varies greatly (2%–25%).^6^ Although its incidence is relatively low, it is almost universally fatal. Thus, it constitutes a very complex therapeutic issue and often requires individualized management.

Many investigations have been performed to discover the contributing factors in the development of SR. Currently, various parameters have been considered as predisposing to SR in the literatures, such as primary location,^7^, ^8^, ^9^, ^10^, ^11^ histological grading,^11^, ^12^, ^13^ tracheostomy timing,^11^, ^14^, ^15^, ^16^ preoperative Radiotherapy (RT),^8^, ^10^, ^15^ post-operative RT,^8^, ^9^, ^17^ preoperative chemoradiotherapy,^18^ T classification,^10^, ^12^, ^17^ N classification,^9^, ^12^, ^19^ pharyngoperistoma fistula,^20^, ^21^ surgical margin,^11^, ^13^ and p53 status.^22^

For head and neck surgeons, a better understanding of these risk factors is critical in patient selection and operative planning. Although several factors have been proposed to be associated with SR in more than one study, conflicting results still exist as to their importance. In fact, which high-risk factors are most significant is still under debate. Although two meta-analyses have been published on this topic,^23^, ^24^ we consider that one study included insufficient number of studies,^23^ and the other focused on the risk factors in those patients who had TL or/and partial laryngectomy.^24^ Thus, we performed a systematic review and meta-analysis in order to investigate the potential risk factors for SR after TL.

## Methods

### Search strategy

A systematic search was performed for all English-language literature published from January 1990 to June 2019. The comprehensive search was performed using the electronic databases PubMed, Web of Science, Ovid, and Cochrane Library. The following keywords were used: laryngeal cancer, total laryngectomy, stomal recurrence, peristomal recurrence, and tracheostomal recurrence. The search strategy was slightly adjusted according to the requirement of different databases. Review studies and bibliographies of other relevant identified investigations were hand-searched to identify additional articles.

### Inclusion and exclusion criteria

All clinical studies were required to meet the following criteria for this study: (1) Proven diagnosis of SR (2) The studies had to report at least one of the risk factors for SR (3) Clinical comparative studies of patients with laryngeal cancer who received TL, either alone or associated with neck dissection (4) Either one of the higher quality or the most recent study was included when two studies were published by the same institution or authors. The following articles were excluded: (1) Partial laryngectomy was performed as the surgery treatment; (2) Letters, comments, expert opinions, reviews, or case reports; (3) Measured outcomes were not clearly presented in the literatures or it was impossible to extract the appropriate data from the articles.

### Data extraction and quality assessment

Two reviewers reviewed each article independently and abstracted data from the articles in accordance with a standardized form. Disagreements were resolved through discussion, and when it the differences were not resolved, a third person made a final decision. The following data were extracted: the authors, publication years, study design, sample size, SR rate, risk factors (tumor location, tumor differentiation, T stage, LNM, paratracheal LNM, preoperative RT, post-operative RT, preoperative tracheotomy – POT), and pharyngoperistomal fistula. There is no widely accepted quality-assessment metric available for case series. Thus, the quality was evaluated using one of the forms issued by The National Institute for Health and Clinical Excellence. This same quality metric has been used in other similar meta-analysis of observational studies.^25^ For quality, scores ranged from 0 (lowest) to 8 (highest) (Table 1). The result of quality assessment for the included studies was as follow: seven studies scored 6, eight studies scored 5, and three studies scored 4.

**Table 1:** Quality assessment for cases series.

1	Case series collected in more than one center (e.g., multicenter study)?
2	Is the hypothesis/aim/objective of the study clearly described?
3	Are the inclusion and exclusion criteria (case definition) clearly reported?
4	Is there a clear definition of the outcomes reported?
5	Were data collected prospectively?
6	Is there an explicit statement that patients were recruited consecutively?
7	Are the main findings of the study clearly described?
8	Are outcomes stratified (e.g., by disease stage, abnormal test results, and patient characteristics)?

### Statistical analysis

We estimated Risk Ratio (RR) and 95% Confidence Interval (CI) and pooled data from across articles to assess the associations between potential risk factors and SR. *Q*-tests and *I*-squared statistics were used to measured statistical heterogeneity between studies. When *p*-value of *Q*-test > 0.1 and *I*^2^ < 50%, a fixed effects model was used; otherwise, a random effects model was applied. The sensitivity analysis was performed by sequentially omitting individual study to check the stability of the result. Publication bias was estimated by visually assessing the asymmetry of Begg's funnel plot.^26^ Finally, Egger's test was used to provide quantitative evidence of publication bias.^27^ Statistical analysis was performed using STATA 12.0 software (Stata Corporation, College Station, Texas, USA) and Microsoft Excel 2010. A *p*-value < 0.05 indicated a statistically significant difference.

## Results

### Study selection and characteristics

**Figure 1:**
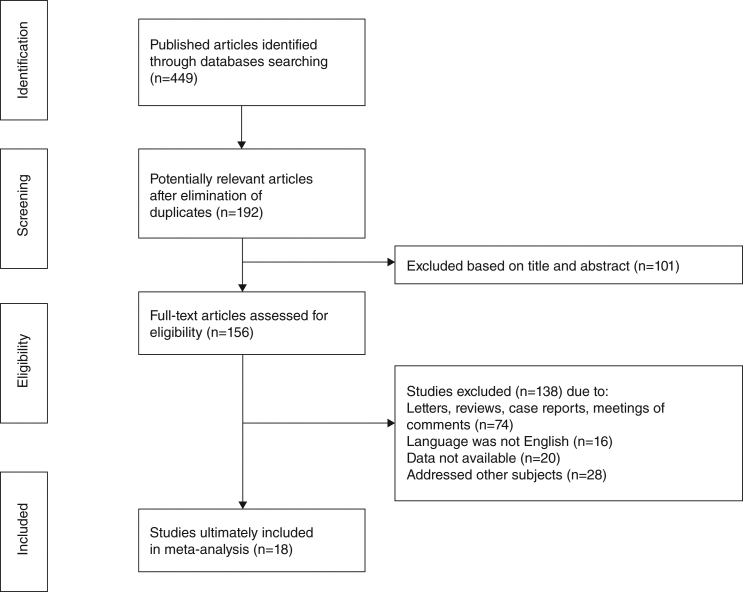
Flowchart showing the studies identified and evaluated during the review.

A total of 449 articles were initially retrieved according to particular keywords, 192 of which were excluded after the initial review of their duplication and language. Approximately a total of 101 studies were excluded after scanning titles and abstracts, leaving 156 articles for full-text assessment. Of these, owing to case reports, reviews, meeting proceedings, conference abstracts and irrelevant studies, 18 studies met the inclusion criteria and finally included in our review.^5^, ^7^, ^8^, ^9^, ^10^, ^11^, ^12^, ^15^, ^17^, ^19^, ^20^, ^21^, ^22^, ^28^, ^29^, ^30^, ^31^, ^32^ The flow diagram illustrating the search and selection process is shown in Fig. 1. The characteristics and methodological quality assessment are shown in Table 2. All the articles included were retrospective studies. The total number of patients included was 6462, ranging from 43 to 1616 patients per study. The rate of SR ranged from 0.8% to 31.3%. SR was diagnosed from 52 day to 5 years after TL.^9^, ^11^, ^21^, ^22^, ^30^, ^32^ Four hundred and sixty-seven patients had preoperative RT,^8^, ^10^, ^15^, ^21^ and 162 patients had post-operative RT.^8^, ^9^, ^15^, ^17^, ^21^ Three studies reported that patients were given postoperative chemotherapy,^11^, ^19^, ^22^ and only one study reported that patients were given preoperative chemotherapy.^8^ Among the 18 studies, 3 were conducted in Greece,^12^, ^20^, ^30^ 2 in China,^11^, ^19^ 2 in the United State,^15^, ^22^ 1 in UK,^5^ 1 in South Africa,^29^ 1 in Brazil,^17^ 1 in Iran,^10^ 1 in Serbia and Montenegro,^9^ 1 in Norway,^28^ 1 in Switzerland,^21^ 1 in Japan,^8^ 1 in Italy,^7^ 1 in Turkey,^32^ and 1 in Spain.^31^

**Table 2:** Main characteristics of studies included.

Author	Country	Design	Number of patients	Follow-up	SR rate	SR time	Patients’ prognosis	Quality score
Brandstorp-Boesen J (2016)	Europe	RT	1616	3.2 years (0–28.3)	0.8%	NA	NA	6
Pezier TF (2013)	Europe	RT	60	16 months (1–91)	8.3%	NA	NA	6
Markou K (2011)	Europe	RT	255	49 months	28.7%	NA	NA	6
Zhao H (2009)	Asia	RT	548	6.3 years (3–13)	8.8%	52 days – 39 months	44 patients with SR died (44/48, 91.7%), the mean survival after SR was 9.2 months (2–11 months)	6
Hassanabadi MS (2007)	Asia	RT	83	15.6 ± 4.8 months	10.8%	NA	10 patients with SR died (7/9, 77.8%)	5
Sartini AL (2007)	South America	RT	47	NA	10.6%	NA	NA	6
Markou KD (2004)	Europe	RT	804	62 months	31.3%	NA	NA	5
Petrovic Z (2004)	Europe	RT	402	NA	9.2%	1–5 years	NA	5
Santoro R (2003)	Europe	RT	464	NA	5.3%	NA	NA	4
Imauchi Y (2002)	Asia	RT	69	47.6 months	8.7%	NA	6 patients with SR died (6/6, 100%), the survival after SR was from 4 to 125 months)	6
Reddy SP (2001)	North America	RT	114	6 years (5–24)	25%	6–11 months	5 patients with SR died (5/5, 100%), the mean survival after SR was within 12 months	5
Fagan JJ (1996)	South Africa	RT	43	NA	16.3%	NA	NA	6
Yotakis J (1996)	Europe	RT	352	NA	6%	1–2 years	NA	5
Yuen AP (1996)	Asia	RT	334	44 months (1–183)	5%	NA	17 patients with SR died (17/17, 100%), the mean survival after SR was 4 months, the longest survival was 16 months	5
Zbären P (1996)	Europe	RT	130	NA	10%	4–30 months	NA	5
Esteban F (1993)	Europe	RT	209	5–12 years	8.1%	NA	NA	4
Hosal IN (1993)	Asia	RT	488	NA	2.7%	3–42 months	NA	5
Rubin J (1990)	North America	RT	444	NA	3.4%	NA	13 patients with SR died (13/15, 86.7%), the mean survival after SR was 9 months (1–22 months)	4

SR, stomal recurrence; RT, retrospective trial; NA, not available.

### Meta-analysis findings

**Figure 2:**
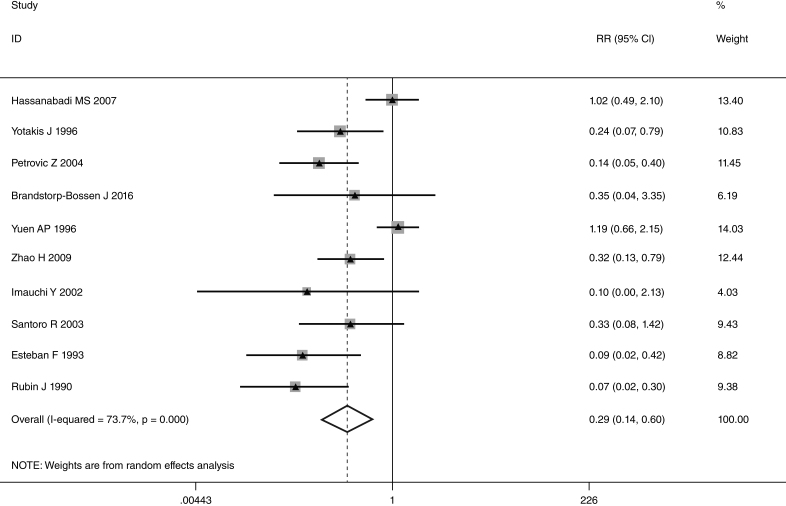
Forrest plot of risk ratio for tumor location (supraglottic vs. subglottic).

**Figure 3:**
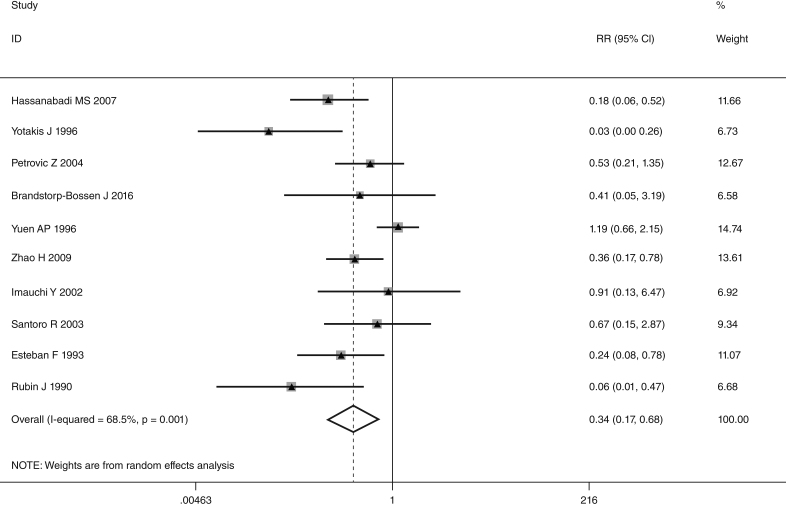
Forrest plot of risk ratio for tumor location (glottis vs. subglottic).

**Figure 4:**
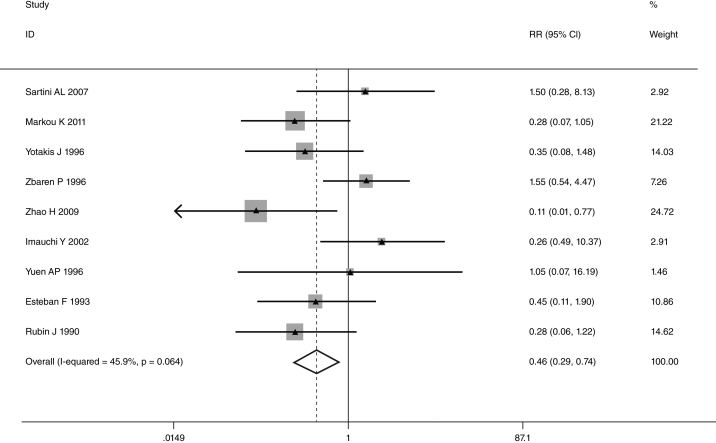
Forrest plot of risk ratio for T stage.

**Figure 5:**
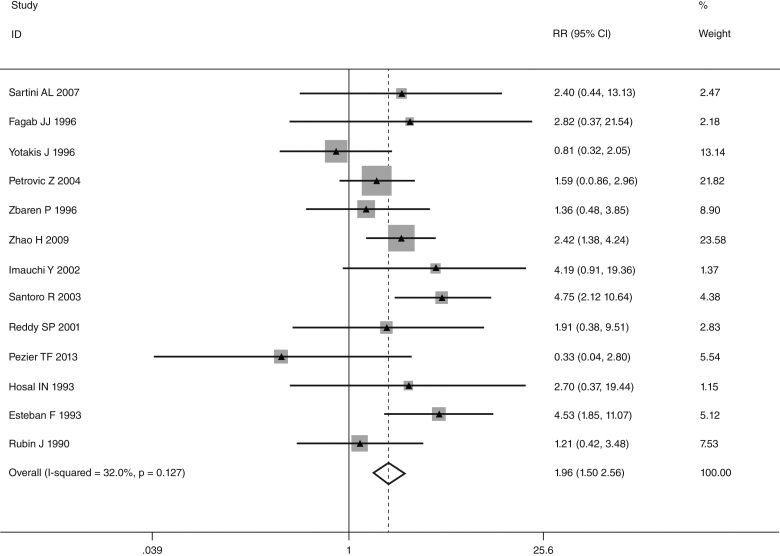
Forrest plot of risk ratio for POT.

This meta-analysis of combinable data was performed to analyze the risk factors for SR, and the main findings are shown in Table 3. Thirteen studies reported data on tumor subsite,^7^, ^8^, ^9^, ^10^, ^11^, ^12^, ^15^, ^17^, ^19^, ^22^, ^28^, ^30^, ^31^ two studies reported data on tumor differentiation,^11^, ^12^ nine studies reported data on T stage,^8^, ^11^, ^12^, ^15^, ^17^, ^19^, ^21^, ^30^, ^31^ eleven studies reported data on LNM,^8^, ^9^, ^11^, ^12^, ^15^, ^17^, ^19^, ^21^, ^22^, ^30^, ^31^ two studies reported data on paratracheal LNM,^8^, ^9^ four studies reported data on preoperative RT,^8^, ^9^, ^10^, ^15^ five studies reported data on post-operative RT,^8^, ^9^, ^15^, ^17^, ^21^ thirteen studies reported data on POT,^5^, ^7^, ^8^, ^9^, ^11^, ^15^, ^17^, ^21^, ^22^, ^29^, ^30^, ^31^, ^32^ and two studies reported data on pharyngoperistomal fistula.^20^, ^21^ When the data were pooled, respectively, tumor subsite (*p* = 0.001, *p* = 0.002) (Figure 2, Figure 3), T stage (*p* = 0.001) (Fig. 4), POT (*p* < 0.001) (Fig. 5) were identified as the risk factors for SR. Specifically, the pooled RRs (95% CIs) were as follows: 0.292 (0.142, 0.600) for tumor subsite (supraglottic vs. subglottic), 0.344 (0.175, 0.676) for tumor subsite (glottic vs. subglottic), 0.461 (0.286, 0.742) for T stage, 1.959 (1.500, 2.558) for POT.

**Table 3:** Statistical results of risk factors for SR after TL.

Risk factors	Number of patients	Number of events	RR (95% CI)	Analytical model	*p*-value
*Tumor location*					
Supraglottic vs. glottic	3905	234	0.842 (0.504, 1.405)	REM	0.509
Supraglottic vs. subglottic	1866	126	0.292 (0.142, 0.600)	REM	0.001
Supraglottic vs. transglottic	856	98	0.459 (0.141, 1.494)	REM	0.196
Glottic vs. subglottic	2475	156	0.344 (0.175, 0.676)	REM	0.002
Glottic vs. transglottic	788	101	0.805 (0.334, 1.939)	REM	0.629
Subglottic vs. transglottic	271	48	1.386 (0.833, 2.305)	FEM	0.209

*Tumor differentiation*					0.559
Well/moderately	573	116	1.435 (0.428, 4.816)	REM	
Poorly	172	18			

*T stage*					0.001
T1 + T2	492	15	0.461 (0.286, 0.742)	FEM	
T3 + T4	1838	213			

*LNM*					0.292
Positive	1009	110	0.780 (0.492, 1.238)	REM	
Negative	1545	105			

*Paratracheal LNM*					0.088
Positive	40	7	1.939 (0.907, 4.143)	FEM	
Negative	429	36			

*Pre-operative RT*					0.240
Yes	162	22	1.778 (0.680, 4.645)	REM	
No	593	115			

*Post-operative RT*					0.552
Yes	467	30	0.802 (0.387, 1.661)	REM	
No	634	46			

*POT*					<0.001
Yes	621	97	1.959 (1.500, 2.558)	FEM	
No	1979	111			

*Pharyngoperistomal fistula*					0.348
Yes	65	21	1.507 (0.640, 3.550)	REM	
No	442	110			

FEM, fixed effects model; REM, random effects model.

### Sensitivity analysis and publication bias

**Figure 6:**
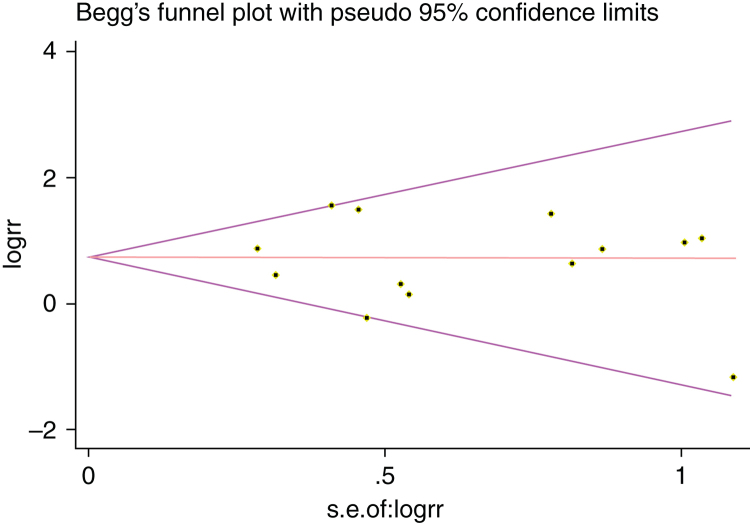
Begg's funnel for the visual assessment of overt publication bias for POT.

**Figure 7:**
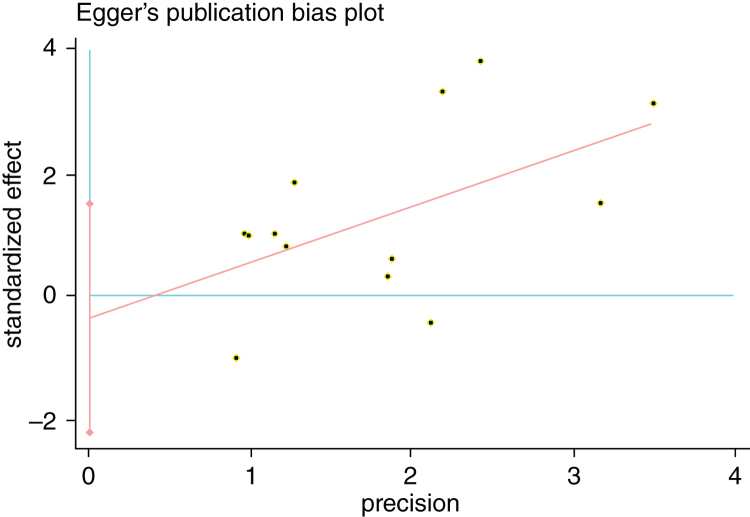
Egger's publication bias plot showed no publication bias for POT.

A single article included in our meta-analysis was deleted each time to reflect the influence of the individual data set to the pooled RR, and the corresponding pooled RR was not materially altered. Begg's funnel plot and Egger's test were performed to access the publication bias of literatures. The shape of the funnel plot did not show any evidence of obvious asymmetry (Fig. 6). In addition, the Egger's test was performed to provide statistical evidence of funnel plot symmetry. Similarly, the outcomes did not reveal any evidence of publication bias (Fig. 7).

## Discussion

Tumor recurrence at the tracheal stomal following TL for laryngeal cancer is a well-recognized phenomenon. Patients with recurrence are commonly diagnosed within the first year after TL but cases have been described to present at 3.5 years.^33^ In previous studies, the occurrence of SR is highly variable. Kowalski et al. proposed it may be explained by the differences in the diagnostic criteria used by each author.^33^ Our results showed that SR rate ranged from 0.8% to 31.3%. We assumed the reason might be the heterogeneity in surgical technique in different periods. Despite refinement of multimodal treatment over the last 20 years, the 5 year survival rate of 40% has remained static since the mid-1980s.^34^ Some studies reported that SR was associated with 90% mortality, and more than 80% patients died in the first two years.^10^ In our study, only six studies evaluated patients’ prognosis,^8^, ^10^, ^11^, ^15^, ^19^, ^22^ and the mortality rate was from 77.8% to 100%. Most of patients died from the progression of the disease. Prognosis for these patients is dismal, despite the development of surgery, chemotherapy and radiation for treatment of SR. In an attempt to establish a more adequate prognosis for these patients, Sisson et al. proposed a classification system for recurrence.^35^ This classification is anatomically based and gives consideration to the site of recurrence but not necessarily its tumor origin or etiology. Patients with types I and II have a median survival of 10 months with 37.5% 1 year survival and 25% 2 year survival. Patients with types I and II have a median survival of 6 months with 40% 1 year survival and 0% 2 year survival. Since current treatment of SR is not ideal, much more attention should be directed to identifying the risk factors to minimize the chance of SR. Despite a large number of articles having been investigated, it is still under debate.

In laryngeal cancer, there is general agreement on the role of subglottic involvement in the development of SR. The occurrence in the range of 38%–44% has been reported in previous studies.^36^ Due to submucosal expansion of tumors, subglottic cancers have a trend to rapid paratracheal tissue involvement. The cricoid cartilage area in subglottic extensions has an extensive blood supply, allowing tumor cells a higher chance of paratracheal direct spreading to infiltrate the thyroid gland and the perilaryngeal soft tissue.^11^, ^15^, ^21^ We found the subglottic region involvement presented a statistical significant trend when compared with supraglottic and glottic tumors. Our result was consistent with previous reports. In addition, patients with subglottic and transglottic involvement presented similar incidence of SR (20.1% vs. 15.3%), and we did not found a significant difference between them.

Some authors considered that tumor size was a significant factor, and it was associated with unfavorable disease free survival.^12^, ^37^ They proposed the possibility that the development of SR increased with tumor size.^38^ Patients with T4 stage were significantly more likely to have SR than those with smaller lesions (T 1–3).^15^ Markou et al. also found large T3 tumor (>4 cm) or small T4 tumor (<2 cm) might have higher SR rate.^12^ Although several authors have reported tumor size contributed to SR, others do not demonstrate any effect.^9^, ^31^, ^39^ Keim et al. reported that no significant correlation in patients with small, intermediate and large cancers.^30^, ^39^ Petrovic et al. identified there was no significant difference between patients with T3 and T4 tumors.^9^ Our results revealed the SR rate in T 1–2 stage and T 3–4 stage was 3.0% and 11.6%, respectively. The patients with higher tumor stage (T 3–4) had significantly higher rate of SR.

At present, nodal status is well established to be an important prognosticator when tumor is located in the larynx.^5^, ^9^ The lymphatic drainage of the larynx has been studied for many years. It is widely acknowledged that the high-risk lymph nodes are often pretracheal and paratracheal lymph node chains. They are rarely palpable and do not initially lead to hoarseness and pain, many patients thus do not have any treatment until the diseases reach an advanced stage, with symptoms of airway obstruction.^40^ Many authors proposed that those LNMs should be regarded as an influential factor for SR.^8^, ^9^, ^41^ This, in turn, seems to influence the incidence of emergency tracheotomy. That is to say, those patients who need POT have higher rates of occult paratracheal LNMs. Considering the pattern of lymphatic metastasis, clearance of the pretracheal and paratracheal lymph nodes should be done to prevent SR. In our study, 1545 patients with N0 disease had 105 recurrences (6.8%), while 1009 patients with N + disease had 110 recurrences (10.9%). However, it was not statistically significant. Simultaneously, we found paratracheal LNM rate was higher in patients with SR (17.5% vs. 8.4%), but a significant correlation between them was not observed.

Emergency tracheostomy prior to laryngectomy has been associated with an increased incidence of SR.^8^, ^31^ The rate of SR in patients who had POT ranged from 8% to 41% in previous studies.^42^ According to the tumor cell implantation hypothesis, dislodged cancer cells have chance to implant in the tissue of the trachea wound.^16^, ^43^ In contrast, some authors have not observed any correlation.^13^, ^44^ They considered this mechanical implantation of tumor cells was less likely, because sufficient margin could be obtained in a properly performed wide field TL, as performed in most hospitals.^8^ Thus, some attention has focused on the other mechanisms, such as the paratracheal LNM, the presence of Delphian lymph node, and the spread of tumor cells throughout the paratracheal tissue and thyroid gland. For example, Rockley et al. found patients who performed POT had more advanced disease and may have a high rate of occult paratracheal LNM, and this increased the risk of SR.^14^ Due to inflammation and fibrosis of tissues around the anastomosis caused by POT, it is difficult to perform the paratracheal lymph node dissection in the clinical practice. Therefore, this also might explain the reason that patients who underwent POT have a higher SR rate. Our results supported that there was a strong correlation between POT and SR.

For the treatment of SR, RT is often used. Some investigations have showed the usefulness of preoperative RT, including the tracheal stoma and the superior mediastinum in the irradiation field.^45^ In addition, several authors found the incidence of SR was significantly lower in the patients who had post-operative RT, and they proposed post-operative RT might have an advantage in preventing recurrence.^9^, ^46^, ^47^ Sartini et al. suggested post-operative RT should be performed when the surgical margins were compromised and minimal, and LNM was demonstrated.^17^ However, the results of our study look controversial: the lack of association between SR and preoperative and post-operative RT. We look forward to obtaining more information from large sample studies for a better comprehension and authentication accuracy in the near future.

The results of this study should be interpreted with caution because of some limitations. First, the retrospective nature of the studies included is clearly a limitation, and these studies are also not the highest-quality evidence, and it might lead to less powerful findings. Second, most of these studies were conducted in European and Asian countries, which may not reflect the real situation. In addition, publication is a major concern for all forms of meta-analysis, and positive findings tend to be accepted by journals, while negative results are often rejected or not even submitted. Although this study does not support publication bias, it should be noted that it could not completely exclude biases. For example, this study was restricted to English-only articles, which probably led to language or cultural bias. However, some authors in their studies suggest that excluding trials published in languages other than English has generally little effect on effect estimates.^48^ Although two meta-analyses have been published on this topic,^47^, ^48^ this study is different from them. We considered that one study included insufficient number of studies,^47^ and the other focused on the risk factors in those patients who had TL or/and partial laryngectomy.^48^ Further research is needed to determine the impact of language restriction.

## Conclusions

In summary, SR after TL continues to be a difficult problem for head and neck surgeons. When SR is present, the prognosis is poor. The etiology of SR after TL is multi-factorial, and identifying high-risk factors is imperative. Our results showed that tumor subsite, T stage, POT were the risk factors associated with higher incidences of SR. This study should be further updated whenever new and strong evidence is available.

## Funding

This work was supported by the Baiqianwan Talent Project (Type C, No. 6) and the 10.13039/501100005047Natural Science Foundation of Liaoning Province (No. 20180530038).

## Conflicts of interest

The authors declare no conflicts of interest.
